# News Items

**Published:** 1918-11

**Authors:** 


### NEWS ITEMS.

Shortage of Platinum.—The War Industries Board asks that every possible scrap of platinum possessed by physicians or stored in unused instruments which can be spared, be turned over to the Government. The need for this metal for war purposes is acute. Accredited representatives of the Red Cross will shortly make a canvas. Beware of turning platinum over to unaccredited individuals. Federal Reserve Banks pay current prices for platinum.

The annual convention of the Southern Medical Association, which was to have been held November 11-14 at Asheville, N. C., has been postponed for one year on account of the influenza epidemic, according to announcement of C. P. Loranz, business manager of the association.

The Government has announced an additional allowance of $8,000 for anti-malaria work for the Fort Worth Zone.

Use of vaccines in combatting or treating Spanish influenza has not gone beyond the experimental stage so far as the United States Public Health Service has been able to learn.

"It must remembered," said Surgeon General Blue in a statement recently, "that several different vaccines are being tried. The reports received do not permit efficacy of these vaccines or their relative merits.

"The Health Service urges the public to remember that there is no specific cure for influenza and that many of the alleged 'cures' and remedies being recommended by neighbors, nostrum venders and others do more harm than good. The chief reliance must be on medical attention, good nursing, fresh air, nutritious food, plenty of water and cheerful surroundings."

State Treasurer J. M. Edwards has just received a check for $42,366.49 which is the share of the State of Texas out of an appropriation of $1,000,000 made by the Federal Government for combatting venereal disease in this country. There was a total appropriation of $5,000,000 made for a period of five years —$1,000,000 to be expended each year. This money is to be expended in Texas under the direction of Dr. H. C. Hall, head of the bureau recently created to stamp out venereal diseases in Texas.

Contract has been let for the Northwest Texas Asylum for the Insane, Wichita Falls. The plant includes nine buildings, the cost to be $343,000. Construction work will begin at once.

Cure of insanity by extracting diseased teeth, removing tonsils and clearing the gastro-intestinal tract, has recently been announced by Dr. Henry A. Cotton, medical director of the New Jersey State Hospital, in a report giving results of eleven years of experimentation on deranged patients.

**U. S. Will Furnish Doctors When Needed.**

Doctors, nurses and medical supplies will be furnished by the United States public health service for communities in Texas that are unable to meet the situation brought about by the influenza epidemic, Maj. C. H. Gardner, in charge of the Government health service in Texas, announces. He also issued a call for doctors and nurses to enroll in the health service. The doctors will be appointed temporary acting assistant surgeons. Liberal salaries and allowances for both doctors and nurses is authorized.

There has been organized at Houston a Public Health Council to assist the military authorities in sanitation work in the Houston zone. It is planned to raise $10,000 to supplement the Government's appropriation for work in that zone.

Population of Texas.—The population of Texas has increased nearly one million since the Federal census of 1910 was taken. Over 400,000 of the gain was due to the stork, while more than 500,000 of the increase was caused by immigration. The Census Bureau at Washington estimated the population of Texas on July 1st, 1918, to be 4,601,279. The population of Continental United States is estimated to be 105,253,000.

Mrs. J. C. Muse of Dallas, Texas, has announced that nine beds have been placed in the American Military Hospital at

Neuilly, France, by the United Daughters of the Confederacy in Texas.

Dr. W. J. Mayo, of the famous Mayo Brothers, announces that a serum has been developed at the Mayo Brothers laboratory which, out of 1,000 cases of influenza treated, has prevented a single case of pneumonia developing. The serum has not been perfected and experiments are continuing.

Three and a half million dollars will be spent in converting Fort Sheridan, Ill., into one of the largest base hospitals in the country and the work of changing the brick barracks into wards already is under way.

The main section of the picturesque north shore army station will be used for the general hospital, which will contain four thousand beds, when completed, six months hence. Sick and wounded American soldiers from France will begin arriving at the hospital within a month.

The Southwestern Flight Surgeons' Association has been organized with headquarters in Dallas, and with members recruited from all the flying fields of Texas, Oklahoma, Louisiana and Arkansas. The officers are Maj. John O. McReynolds, Camp Dick, president, and Capt. Frederick C. Lewitt, Rich Field, secretary.

